# Association Between Vitamin D Deficiency and Duration of Hospital Stay, Pediatric Intensive Care Unit Stay, and Ventilation; Pediatric Risk of Mortality Score; and Rate of Readmission: A Prospective Observational Study

**DOI:** 10.7759/cureus.10322

**Published:** 2020-09-09

**Authors:** Sarthak Das, Kiran Kumar M, Niranjan Biswal, Narayanan Parameswaran, Nivedita Nanda

**Affiliations:** 1 Paediatrics, All India Institute of Medical Sciences, Mangalagiri, IND; 2 Paediatrics, Jawaharlal Institute of Postgraduate Medical Education and Research, Puducherry, IND; 3 Biochemistry, Jawaharlal Institute of Postgraduate Medical Education and Research, Puducherry, IND

**Keywords:** children, paediatric intensive care unit (picu stay), prism score, ventilation, vitamin d deficiency

## Abstract

Introduction

The study aims to evaluate the association between a deficiency of Vitamin D level with the duration of hospital stay, pediatric intensive care unit (PICU) stay, and ventilation; the pediatric risk of mortality (PRISM) score, and the rate of readmission.

Materials and methods

This prospective observational study was conducted from November 2014 to October 2015, and the study population consisted of children admitted to the pediatric intensive care unit (PICU) in a tertiary care hospital of Puducherry. After measuring vitamin D levels, children were allotted into three categories depending on their serum 25(OH)D levels as the sufficient group (25[OH]D level ≥ 30 ng/mL), insufficient group (25[OH]D level = 20 - 29.9 ng/mL), and deficient group (25[OH]D level < 20 ng/mL). Among these three groups, the duration of hospital stay, PICU stay, and ventilation; the PRISM score, and the rate of readmission were compared.

Results

A total of 522 patients were included in the study. Based on their 25(OH)D level, 222 patients (42.5%) were in the sufficient category, 153 patients (29.3%) were in the deficient category, and 147 patients (28.2%) were in the insufficient category. Vitamin D deficiency state is not statistically significantly associated with the duration of hospital stay (P = .84), duration of PICU stay (P = .69), duration of ventilation (P = .48), PRISM score (P = .63), and rate of readmission (P = .91).

Conclusions

Longer hospital stay, prolonged PICU stay, longer duration of ventilation, and higher PRISM III score were independent risk factors for higher mortality in the PICU. However, lower vitamin D levels are not statistically significant to predict mortality among the study population.

## Introduction

Vitamin D has a significant part in calcium equilibrium and skeletal system development in the human body. Immune cells like B cells, T cells, and antigen-presenting cells have receptors for vitamin D, so these immune cells produce vitamin D metabolite. The immune response is significantly regulated by vitamin D, and there is increased susceptibility to infection and autoimmune disease among vitamin D-deficient patients [1]. Other than classical bone manifestations, children with vitamin D deficiency (VDD) are susceptible to systemic involvement like the central nervous system, cardiovascular system, respiratory system, and immune system [2-5]. These organ systems have a key role in the process of pathogenesis, clinical features, complication, and recovery from the state of critical illness. Therefore, VDD is a crucial factor for morbidity and mortality in the pediatric intensive care unit (PICU) [6].

In some studies, VDD has a relevant association with severe illness at presentation, increased the requirement of inotropes, the requirement and duration of mechanical ventilation, the duration of hospitalization, and mortality rate [7-14]. In contrast, these associations were not found to be relevant in other studies [15-16]. Whether the administration of vitamin D influences the outcome of patients, is a silent observer, or is a clinical indicator of disease severity are different problematic statements found due to the lack of availability of evidence among children and limited interventional studies in India. There was no significant data associating VDD with clinical outcomes like the duration of PICU stay, duration of hospital stay, pediatric risk of mortality (PRISM) score, the requirement for ventilation, and the duration of ventilation in the Indian scenario [15-17].

Therefore, we conducted a prospective observational study to look for an association between VDD and the duration of hospital stay, the duration of PICU stay, the duration of ventilation, the PRISM score, and the rate of readmission.

## Materials and methods

We conducted this prospective observational study from November 2014 to October 2015, and the study population consisted of children admitted to the PICU of our tertiary care hospital.

All children aged between one month and 12 years who were admitted to the PICU were included in the study. All cases of rickets (either a known previous case or a case diagnosed for the first time after hospitalization) and cases who obtained vitamin D supplementation within the last 30 days of hospital admission, cases admitted in the PICU only for monitoring purposes following a procedure or surgery and during anti-snake venom administration were excluded from the study.

Cases were enrolled after obtaining informed consent from parents, caretakers, or guardians. Parents, caretakers, or guardians were interviewed regarding the detailed chief concern, duration of sun exposure by outdoor activities, and history of vitamin D supplementation of patients, and data were collected.

As early as possible (maximum limit up to 24 hours) after being admitted to the PICU, 2 mL of venous blood was collected for measuring 25(OH)D levels. The blood sample was allowed to clot at room temperature and centrifuged for the preparation of serum. The prepared serum sample was frozen at -80°C, stored, and then used for measuring the 25(OH)D level by the enzyme-linked immunosorbent assay method, which was developed by Calbiotech (El Cajon, CA) and procured by BioDiagnosis (River Falls, WI).

After collecting patient data and measuring vitamin D levels, children were separated into three categories, depending on their serum 25(OH)D levels, as the sufficient group (25[OH]D level ≥ 30 ng/mL), insufficient group (25[OH]D level = 20-29.9 ng/mL), and deficient group (25[OH]D level < 20 ng/mL) [18].

Statistical analysis

For a description of the data, statistical mean (± SD), frequencies (number of cases), and percentages were used as per requirements. The Kolmogorov-Smirnov test was used to check the normal distribution of data. The student’s t-test was used to compare the independent quantitative variables between the study groups when normally distributed. For comparison of non-normally distributed quantitative data, a Mann-Whitney U test was used. One-way analysis of variance with post-hoc Tukey’s test was used to compare more than two groups. A chi-square test was used for comparing categorical data. Fisher’s exact test was used for all 2 x 2 tables.

A binary logistic regression analysis was performed to find the significant predictors of the outcome by taking survival as the dependent variable, and variables like serum 25(OH)D levels, duration of PICU stay, hospital stay, and duration of ventilation are independent risk factors for mortality.

All statistical calculations were done using the computer programs Microsoft Excel 2007 (Microsoft Corporation, Redmont, WA) and SPSS Statistics for Windows, Version 17.0 (SPSS Inc., Chicago, Il). A P-value of less than .05 was considered statistically significant.

## Results

A total of 580 patients were admitted to the PICU from November 2014 to October 2015, and 522 patients met the criteria for inclusion in the study.

Two-hundred twenty-two patients (42.5%) were in the sufficient group, 153 patients (29.3%) in the deficient group, and 147 patients (28.2%) in the insufficient category. A total of 435 cases were alive, and the rest (87 cases) were dead. Of all 87 death cases, 29 cases (33%) belonged to the sufficient group, 25 cases (28%) to the insufficient group, and 33 cases (37%) to the deficient group. Of all study children, 48.9% were infants in the age group of one month to one year, of which 34.9% were deficient, 27.8% were insufficient, and 32.7% had healthy 25(OH)D values.

The mean serum 25(OH) D levels in these different categories are represented in Table 1.

**Table 1:** Mean 25(OH)D levels among groups Abbreviation: IQR, interquartile range; SD, standard deviation

	Sufficient	Insufficient	Deficient
Mean (ng/mL)	39.62 (IQR; 33.80–54.28)	24.54 (SD ± 4.147)	13.37 (SD ± 4.8)

In our study population, 285 cases (54.6%) were boys and 237 (45.4%) were girls. The most common cause of PICU admission (27.2%) was respiratory diseases. Of the 153 cases with a deficient level of 25(OH)D, 28.1% had a respiratory illness, 19% had central nervous system (CNS) disease, and 13.1% had cardiovascular disease (Table 2).

**Table 2:** System-wise distribution of cases Abbreviation: CNS, central nervous system

	Vitamin D Status				
System involved	Sufficient N (%)	Insufficient N (%)	Deficient N (%)	Total N (%)	P-value
Respiratory	63 (28.4)	36 (24.5)	43 (28.1)	142 (27.2)	0.41
Cardiovascular	30 (13.5)	21 (14.3)	20 (13.1)	71 (13.6)	
CNS	46 (20.7)	35 (23.8)	29 (19.0)	110 (21.1)	
Gastrointestinal	17 (7.7)	10 (6.8)	8 (5.2)	35 (6.7)	
Renal	8 (3.6)	6 (4.1)	9 (5.9)	23 (4.4)	
Hematological	17 (7.7)	7 (4.8)	3 (2)	27 (5.2)	
Others	40 (18.0)	32 (21.8)	41 (26.8)	113 (21.6)	
Total (%)	222 (42.5)	147 (28.2)	153 (29.3)	522 (100)	

The mean duration of hospital stay in the deficient group was 206.76 hours (SD, 164.11 hours). The insufficient group’s mean hospital stay was 218.61 hours (SD, 245.16 hours). The sufficient 25(OH)D level group’s mean hospital stay was 210.53 hours (SD, 142.02 hours). The difference in the incidence of hospital stay is not statistically significant (P = .84; Table 3).

**Table 3:** Duration of hospital stay, duration PICU stay (measured in hours), and PRISM III score in PICU admitted children Abbreviations: PICU, pediatric intensive care unit; PRISM, pediatric risk of mortality; SD, standard deviation

Variables	Group	Mean ± SD	P-value	
Hospital stay in hours	Normal	210.53 ± 142.02	0.84	
	Insufficient	218.61 ± 245.16		
	Deficient	206.76 ± 164.11		
PICU stay in hours	Normal	142.39 ± 121.19	0.69	
	Insufficient	155.03 ± 235.25		
	Deficient	140.15 ±136.57		
PRISM III score	Normal	15.59 ± 7.14	0.63	
	Insufficient	16.26 ± 8.94		
	Deficient	16.29 ± 8.67		

Regarding stay in the PICU, the median duration of stay in the PICU is 140.15 hours (SD, 136.57 hours) in the deficient group, 155.03 hours (SD, 235.25 hours) in the insufficient group, and 142.39 hours (SD, 121.19 hours) in the sufficient group; these differences were not statistically significant (P = .69).

The PRISM III score to predict the expected mortality of PICU admitted patients was 16.29 (SD, 8.67) in the 25 (OH)D deficient group, 16.26 (SD, 8.94) in the insufficient group, and 15.59 (SD, of 7.14) in the sufficient level group, which were not statistically significant (P = .634; Table 3).

Vitamin D levels and the PRISM III score were negatively correlated as measured by Pearson correlation r=-0.04, and the correlation was not statistically significant (P = .338).

Only 13 of 522 patients were readmitted, of which 30.8% were from the deficient group and 23% were from the insufficient group (Table 4). Together, both contributed to 53.8% of the cases readmitted and 46.2% of cases were from the sufficient group.

**Table 4:** 25(OH)D status with rate of readmission and ventilation requirement in the PICU Abbreviation: PICU, pediatric intensive care unit.

25(OH) D Status				
Readmission	Sufficient n (%)	Insufficient n (%)	Deficient n (%)	P-value
No	216 (96.8)	144 (98.0)	149 (97.4)	.91
Yes	6 (2.7)	3 (2.0%)	4 (2.6)	
Total (%)	n =222 (42.5%)	n=147 (28.2%)	n=153 (29.3%)	
Ventilation				
No	116 (52.3)	70 (47.6)	69 (45.1)	.48
Yes	106 (47.7)	77 (52.4)	84 (54.9)	
Total (%)	n =222 (42.5)	n=147 (28.2)	n=153 (29.3)	

The ventilation requirement in the PICU was higher in the 25(OH)D deficient group (54.9%) compared with the insufficient group (52.4%) and the sufficient group (47.7%). There was no statistical significance in our comparisons (P = .48). The association between vitamin D status with serum calcium and phosphate level is presented in Table 5.

**Table 5:** Serum calcium and phosphate levels associated with 25(OH)D Abbreviations: Ca, calcium, PO4, phosphate; SD, standard deviation.

Variables	Mean Vitamin D Status			P-value
	Sufficient (±SD)	Insufficient (±SD)	Deficient (±SD)	
Ca	9.24 ±1.23	10.33 ± 9.78	9.06 ± 1.46	.14
PO_4_	4.04 ± 0.98	4.22 ± 1.37	4.13 ± 1.01	.44

In univariate analysis, the variants found to be significantly associated with mortality were selected for multivariate analysis to determine the independent predictors of mortality. In multi-logistic regression analysis, mortality was not associated with vitamin D levels, but the duration of PICU stay, hospital stay, and duration of ventilation were associated with mortality (Table 6).

**Table 6:** Multi-logistic regression for mortality Abbreviations: Ca, calcium; CI, confidence interval; EXP(B), PICU, pediatric intensive care unit; PO4, phosphate; PRISM, pediatric risk of mortality

	Logistic Regression: Mortality(R^2^=0.46)			
Variables	Odds Ratio	95% CI for EXP(B)		P-value
		Lower	Upper	
Vitamin D levels	0.996	0.974	1.005	.192
PICU Stay	1.024	1.007	1.042	< .01
Hospital Stay	0.98	0.975	0.985	< .01
Ca	0.922	0.706	1.203	.55
PO_4_	1.022	0.757	1.38	.88
Duration of ventilation	1.02	1.01	1.031	< .01
Ventilation	1.007	0.967	1.048	.74
PRISM III score	0.28	1.08	1.12	< .01

## Discussion

The prevalence rate of VDD observed in the present study is 29.3%, which is a smaller percentage than those reported by previous studies, which ranged from 30% to 85.7% [7,10,19-23]. The difference in the prevalence of VDD might be due to the duration of outdoor activity with quality of sun exposure, the exposed surface area of the skin during outdoor activity, pigmentation of the skin, nutritional status, and various genetic factors [7].

In the present study population, 48.9% were infants (i.e., aged one month to one year). The mean 25(OH)D level was lowest in the one month to one year age group despite children in this age group having the protection of breast milk feeding. This may suggest a case of pre-existing VDD in lactating mothers of study participants.

Sankar et al. found a median serum vitamin D level of 5.8 ng/mL (IQR: 4-8) in 25(OH)D deficient patients and a median 25(OH)D level of 22.5 ng/mL (IQR 16.4-31.3) [20]. The median value of serum vitamin D of the present study was 13.37 ng/mL (SD ± 4.8 ng/mL), which was higher than the study done in Delhi. In the present study, there was a higher level of median serum vitamin D (28 ng/mL) among one to 11-year-old children than in a USA study [24]. The wide variation in the median level of vitamin D level in different countries is probably due to regional trends in nutritional status, sun exposure duration, skin pigmentation, climatic changes, dietary habits, and underlying medical illnesses between study populations.

In the present study, the distribution of medical involvement was similar to the distribution reported by Venkatram et al., with respiratory cases as the predominant cause of admission involving 18.8% cases, 12.5% central nervous system (CNS) cases, and 10.5% as gastrointestinal (GI) cases [25].

The mean duration of hospital stay is aligned with those reported by Amrein et al. in Austria, in 2014, in their study of critically ill adult patients who found no significant change in the duration of hospital stay between vitamin D3 supplementation and the placebo group [26]. However, a meta-analysis conducted by Zhang et al. found that the duration of hospital stay was prolonged in hypovitaminosis D in 2014 [27].

The present study group found very little difference in the mean duration of PICU stay between study groups, but there was an increased duration of PICU stay in the 25(OH)D deficient group by approximately 72 hours as compared to the sufficient group, which was conducted by another study in India [20]. The study by McNally reported a mean PICU stay of four days, which was shorter than our findings [8]. The difference in duration is probably due to individual clinical case profiles, diagnosis at the time of admission, age of the patient, the nutritional profile of the patient, and genetic factors. Also, the duration of PICU stay was associated with the duration of ventilation and severity of illness. However, in the present study, PICU severity scores and duration of ventilation were not associated with vitamin D levels; therefore, the same was also replicated in the duration of the PICU stay.

The mean duration of mechanical ventilation in our study was three days, which was similar to the 3.5-day mean reported in a Canadian study but briefer than the nine-day mean reported by a north Indian study [1,3]. The discrepancies may be due to adequate sunlight exposure during outdoor activity, independent of the dietary profile. Moreover, the children in our study population may have good nutritional status, which led to shorter disease duration, better recovery, and shorter duration of mechanical ventilation [1,3]. The duration of mechanical ventilation was significantly associated with mortality in the vitamin D deficient group.

The mean PRISM III score was 16.1 (SD, 8.14) in our study. Vitamin D levels and PRISM III score are negatively correlated as measured by the Pearson correlation r = -.04 and statistically not significant (P = .33), which means that the lower the vitamin D levels, the higher the PRISM III score will be, and the observed mortality is the same as those reported in other studies [28]. The median PRISM-III score was 5 (IQR, 0-11.5), and it was inversely correlated with 25(OH)D level (r = 20.23, P < .0001) reported by McNally et al. [4]. In both studies, the PRISM III score was negatively correlated with 25(OH)D levels, but the score was higher in our study as compared to the results of a study done in Canada [20]. This difference is probably due to a discrepancy in the clinical status of patients, especially the vitals of the patient and the severity of disease at the time of presentation to the PICU. A similar study from northern India had reported that the PRISM score, sequential organ failure assessment score, and duration of PICU stay were not associated with 25(OH)D deficiency [15].

Lower serum 25(OH)D levels had not been consistently associated with increased mortality or predictive mortality score in children [20]. The 90-day mortality did not differ among patients with or without VDD (28.3% vs. 28.5%, P = .78) in a study from Finland [29].

The events of hypocalcemia were not significantly observed in patients in a VDD state in our study. The cases with at least one hypocalcemia event had characteristically low vitamin D levels than compared to cases with healthy calcium status seen in a Canadian study [4].

Longer hospital stay (P ≤ .01), prolonged PICU stay (P ≤ .01), longer duration of ventilation (P ≤ .01), and PRISM III score (P ≤ .01) were independent risk factors for higher mortality in the PICU. However, lower vitamin D levels are not statistically significant in predicting mortality in multiple logistic regression analysis (P = .19). Contrary to our findings, mortality was not associated with the PRISM score or prolonged PICU stay in a study reported from Haryana, India [30].

On multivariate analysis, the association between length of ICU stay and VDD remained significant, even after adjusting for key baseline variables, diagnosis, illness severity, pediatric logistic organ dysfunction, and need for fluid boluses, ventilation, inotropes, and mortality (adjusted mean difference 3.5 days; 95% CI: 0.50-6.53; P = .02) [20].

Septic shock, multiorgan dysfunction syndrome, duration of mechanical ventilation, blood culture positivity, hypocalcemia, and length of PICU stay were not modified by the presence of 25(OH)D deficiency in a study from north India [24].

Hypovitaminosis D and its association on mortality and morbidity (increased length of stay, positive blood culture, duration of mechanical ventilation, infections) show a positive correlation. Even though causality was not established, studies conducted globally have supported this association [7,21,25].

The strengths of our study include an adequate sample size for analysis. The duration of the study was one full year, so the factors responsible for vitamin D metabolisms like climate shift and outcome of sunlight exposure were minimized. This study described the prevalence of vitamin D among critically ill children admitted to the PICU with highly deranged physiology of the vital organs. A limitation of our study was that the real picture of vitamin D prevalence at the community level could not be deduced, as the site of the study was in the PICU of a tertiary hospital.

## Conclusions

Our study compared different variables like duration of PICU stay, hospital stay, culture positivity, and calcium and phosphate with vitamin D levels and analyzed to see the correlation to vitamin D status. A longer hospital stay, prolonged PICU stay, longer duration of ventilation, and a higher PRISM III score were independent risk factors for higher mortality in the PICU. However, lower vitamin D levels were not significant to predict mortality among the study population.
